# Nurses’ experiences of using falls alarms in subacute care: A qualitative study

**DOI:** 10.1371/journal.pone.0287537

**Published:** 2023-06-22

**Authors:** Julie Considine, Debra Berry, Maureen Mullen, Edmore Chisango, Melinda Webb-St Mart, Peter Michell, Peteris Darzins, Leanne Boyd

**Affiliations:** 1 School of Nursing and Midwifery and Centre for Quality and Patient Safety Research in the Institute for Health Transformation, Deakin University, Geelong, Victoria, Australia; 2 Centre for Quality and Patient Safety Research–Eastern Health Partnership, Eastern Health, Box Hill, Victoria, Australia; 3 Eastern Health, Box Hill, Victoria, Australia; 4 Eastern Health Clinical School, Monash University, Clayton, Victoria, Australia; The University of York, UNITED KINGDOM

## Abstract

Bed and chair alarms have been included in many multifaceted falls prevention interventions. None of the randomised trials of falls alarms as sole interventions have showed significant effect on falls or falls with injury. Further, use of bed and chair alarms did not change patients’ fear of falling, length of hospital stay, functional status, discharge destination or health related quality of life. The aim of this study was to explore nurses’ experiences of using bed and chair alarms. A qualitative descriptive study using semi-structured interviews with a purposive sample of 12 nurses was conducted on a 32-bed Geriatric Evaluation and Management ward in Melbourne, Australia. Participants were interviewed between 27 January and 12 March 2021.Transcribed audio-recordings of interviews were analysed using inductive thematic analysis. NVIVO 12.6 was used to manage the study data. Three major themes and four subthemes were constructed from the data: i) negative impacts of falls alarms (subthemes: noisy technology, imperfect technology), ii) juggling the safety-risk conflict, and iii) negotiating falls alarm use (subthemes: nurse decision making and falls alarm overuse). Nurses’ experience of using falls alarms was predominantly negative and there was tension between falls alarms having limited impact on patient safety and risks associated with their use. Nurses described a need to support nurse decision making related to falls alarms use in practice and policy, and a desire to be empowered to manage falls risk in other ways.

## Introduction

Reducing falls and, especially injurious falls, in hospital patients is a national and international patient safety priority. The traditional approach to falls prevention in hospitals has been risk assessment, and implementation of falls prevention strategies. However, the effectiveness of falls risk assessments or specific falls prevention strategies is unclear with heterogeneity in study design and results [1]. There are over 26 validated tools used to assess falls risk in older persons in a range of settings, many of which do not have high predictive validity for differentiating high and low fall risks [2]. A recent cluster randomised controlled trial showed that removing a falls risk screening tool (but with continued risk assessment and selection of prevention interventions) did not adversely affect falls or falls with serious injury [3]. However, the intervention significantly reduced the time per patient reported by staff to complete falls prevention paperwork [3], calling into question the clinical utility and effectiveness of falls risk assessment tools.

Falls prevention strategies have been an area of focus for many years, however most studies report falls prevention bundles of care making the evidence for specific interventions difficult to ascertain. A systematic review and meta-analysis of 283 randomised controlled trials of falls prevention interventions for older persons in all settings (home, community, hospital) showed that exercise alone and various combinations of interventions were associated with lower risk of injurious falls compared with usual care [4]. However, only 51 of these studies were in hospital settings and only 16 hospital studies tested a single intervention against usual care [4]. The largest multisite randomised controlled trial of falls risk assessment and one or more interventions from a bundle of falls prevention strategies (falls alert sign, bathroom supervision, walking aids within reach, toileting regimen, low-low bed, bed/chair alarm) in Australian medical and surgical ward patients, failed to showed a significant difference in falls or injuries resulting from falls [5].

Bed and chair alarms have been included in many multifaceted falls prevention interventions [4–6] but there are few studies of the effect of bed and chair alarms in isolation for hospital populations. None of the randomised trials of falls alarms as sole interventions have shown significant effect on falls [7,8] or falls with injury [8] and use of bed and chair alarms did not change patients’ fear of falling, length of hospital stay, functional status, discharge destination or health related quality of life [8]. Further, in a cluster randomised controlled trial of an educational intervention for nurses, falls alarm use increased in the intervention wards, however there were no significant differences in falls, falls with injury or use of physical restraint [9]. It should be noted that the authors cautioned that the study was underpowered to detect the primary end point of number of falls [9].

Strategies aimed at falls prevention in hospital patients are costly. An Australian multisite study estimated the annual opportunity cost of falls prevention efforts across six health services was over $46 million AUD and extrapolated to a national level, over $590 million AUD per year [10]. Approximately 11% of these costs were attributed to falls prevention alarms [10]. Disinvestment in falls alarms is possible. A study reporting on a policy change to reduce falls alarms in acute care wards and eliminate falls alarms in rehabilitation wards showed that falls alarm use went from 7.1% to 1.8% in acute care (p<0.001) and 10.5% to 0% in rehabilitation (p<0.001) [11]. The policy and practice change also resulted in a small increase in implementation of confounding falls reduction strategies in the rehabilitation wards but no change in falls prevention strategies used in the acute care wards [11]. The study was not sufficiently powered to determine the impact of disinvestment on the frequency of falls [11].

The aim of this study was to explore nurses’ experiences of using bed and chair falls alarms. For the purposes of this paper, the term ‘falls alarms’ includes both bed and chair alarms.

## Materials and methods

### Design

A qualitative descriptive approach was used [12] with study data collected using semi-structured interviews and analysed using inductive thematic analysis. Ethics approval for the conduct of this study was received from the Human Research Ethics Committee at Eastern Health (E20-023-69030). This study is reported according to the Consolidated Criteria for Reporting Qualitative Research [13].

### Setting

The setting for this study was one 32 bed Geriatric Evaluation and Management ward from a health service in Melbourne, Australia. The nurse-to-patient ratios on the study ward were one nurse to five patients on a morning shift, one nurse to six patients on an afternoon shift and one nurse to ten patients overnight. There were 43 nurses on the study ward roster. Of these 16% were registered nurses on morning shifts, 14% on evening shifts and 12.5% overnight, and the remainder were enrolled nurses. Registered nurses are Bachelor prepared and enrolled nurses are Diploma prepared and work under the supervision of registered nurses. The average length of patient stay on the study ward was 21.2 days.

Falls data from 1 July 2019–30 June 2020 provided context for this study. During this time there were 222 falls in 110 patients: the median number of falls per patient was one (range 1–24 falls per patient). At the patient level, 52.7% (n = 58) were females and the median age was 86 years (interquartile range 80 to 89). The median subacute care length of stay in total was 22 days (interquartile range 14 to 40 days). Of the 110 patients in this study, 2 died during their subacute care admission. The most common location of falls was patient rooms (72.1%, n = 160), the most common activity at the time of the fall was standing or walking (25.2%, n = 56) and two-thirds of falls were unwitnessed (64%, n = 143). Various falls prevention strategies were in place at the time of the fall, including falls risk alert sign over the bed (90.1%, n = 200), direct bathroom supervision (82.9%, n = 184), falls alarm (61.7%, n = 137) and lo-lo bed (hospital bed that can be lowered to floor level) (30.6%, n = 68). Most falls (96.8%, n = 213) resulted in no or minimal physical harm.

### Participants

Purposive sampling was used to recruit participants. All nursing staff permanently employed on the study ward were invited to participate in a semi-structured interview during ward meetings and in-service education sessions. Study recruitment was managed by one researcher (JC) who had no line management or operational responsibilities. Written informed consent was obtained from all participants.

### Data collection

All interviews were conducted by one female researcher, who is a PhD-qualified registered nurse with experience in qualitative research and clinician interviews (JC), between 27 January and 12 March 2021. The interviewer holds a joint academic-industry professorial appointment at the same health service but has no direct-line management or clinical interaction with the participants. Eleven interviews were face-to-face, and the remaining interview was conducted by telephone: they were audio-recorded and ranged from 11 to 18 minutes in duration. The semi-structured interview guide was developed following literature review and consultation with the research team, health service Comprehensive Care Clinical Risk Governance Committee, and health service consumer representatives. The interview guide is presented in Table 1.

**Table 1:** Interview guide.

• Do you regularly use falls bed or chair alarms? *Probe*: *why*? *Can you tell us a bit more about that*? • Do you feel that the falls bed or chair alarm makes patients less likely to fall? *Probe*: *YES*: *how*? *Can you explain a little more*? *NO*: *what are the things you think make patients safer and reduce their risk of falling*? Do the falls bed or chair alarm cause you any problems? *Probe*: *can you explain a little more*? • What impact do you feel removing the falls bed and chair alarms will have on falls? On your day-to-day work?

Data were transcribed and coded following each interview. At the conclusion of every third interview, the codes were reviewed to determine whether new information was being obtained, or whether information obtained from preceding interviews was confirmed, thus indicating data saturation [14]. Data saturation was apparent after ten interviews so two more interviews were undertaken to confirm that no new information was evolving. This methodological decision was based on a study suggesting that data saturation was achieved within 12 interviews when participants were asked the same interview questions [15].

### Data analysis

The interviews were transcribed verbatim using a professional transcribing service. Inductive thematic analysis was undertaken using six steps from the framework developed by Braun and Clarke [16,17]: familiarisation with the data; generating initial codes; searching for themes; reviewing themes; defining and naming themes and producing the report. A single researcher (JC) checked the transcripts for accuracy against the audio files, entered them into NVIVO software, and after close reading and re-reading, performed the initial coding. An open coding process was used, so codes were not determined *a priori*, but developed and modified during the coding process [16,17]. A second researcher (DB) reviewed the codes and through an iterative process and discussions, subthemes and themes were identified from the data. Differences of opinion during initial coding were resolved by re-reading the interview transcripts, listening to the interviews and discussion. The themes, subthemes, codes, and transcript content for each code were organised into tables, circulated to the research team, and discussed at fortnightly to monthly team meetings where themes and subthemes were refined to the final versions presented in this paper.

The tenants of rigor of qualitative research are credibility, transferability, dependability, and confirmability leading to trustworthiness [18]. The systematic development of the interview guides established credibility, and dependability was obtained by the sound methodological approach undertaken. Conformability was established by using examples from the interviews to ensure that nurses’ voices were represented. A reflexive approach to thematic analysis requires researchers to question their assumptions, highlights researchers’ skills as resources, and requires researchers’ reflexive engagement with the data in its interpretation [17]. The research team was diverse and comprised nursing researchers, experts in subacute care nursing, ward and executive management, a clinical risk manager, and a consumer. Each member of the team therefore brought a specific perspective to the conversations regarding data analysis and theme and subtheme generation. Given the pragmatic nature of this work, the peer-to-peer conversations and meeting notes served a similar purpose to that of a reflexive journal, and enabled tracking of methodological discussions and decisions. Member checking (return of transcripts to participants) did not occur to decrease participant burden. Example interview data and their respective codes, subthemes and themes are shown in Table 2.

**Table 2:** Examples of codes, subthemes and themes.

Data	Code	Subtheme	Theme
“it is very distracting if you’re trying to do a medication round or you’re trying to do a dressing and so you need to be concentrating on what you’re doing. You know you’ve got a patient that’s a high falls risk and it’s likely them that’s making your pager go off. It is very distracting” (Nurse 9—EN)	Interrupts nursing care	-	Juggling the safety-risk conflict
Probably about 80 per cent of the time the patients actually aren’t getting up. They’ve just shifted in their seat or their position and it’s gone off. … (Nurse 1—EN)	False alarms	Imperfect technology	Negative impacts of falls alarms
…if it’s something like ongoing delirium or something and they just keep getting up and doing things that might be a risk to themselves then the bed chair alarm goes on (Nurse 1—EN)	Patient characteristics	Nurse decision making	Negotiating falls alarm use

## Results

A convenience sample of 12 nurses from the study ward were interviewed. Two participants were male, and participants’ levels of appointment included enrolled nurses (ENs) (n = 4), registered nurses including clinical nurse specialists (n = 5) (combined for anonymity) (RNs) and associate nurse unit managers (n = 3) (ANUMs). Participants had between six months and fourteen years clinical experience within the study setting (0 to 3 years, n = 3; 4 to 6 years, n = 3; 7 to 10 years, n = 0; 11 to 14 years, n = 5; and no response, n = 1).

**Fig 1:**
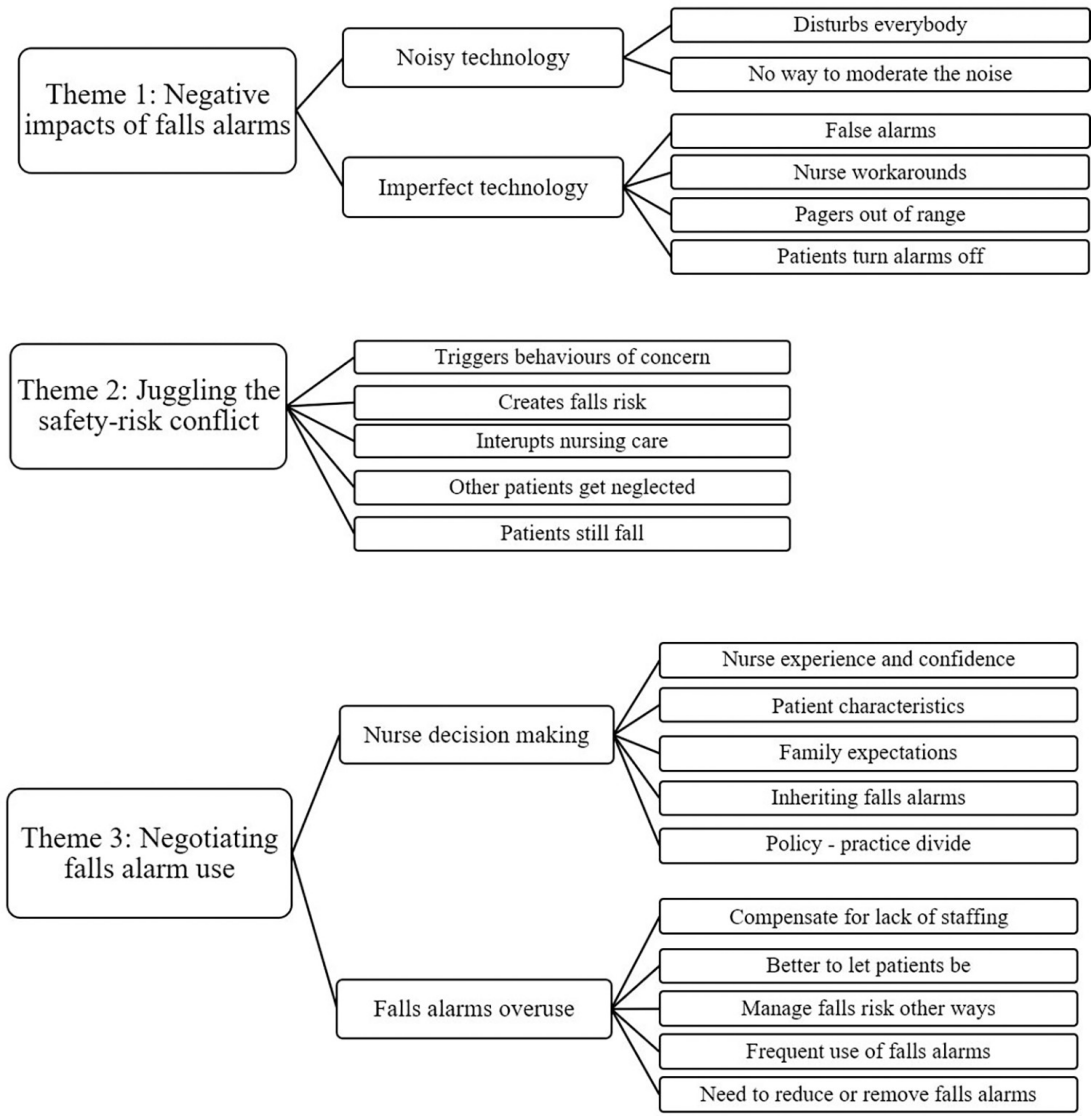
Concept map of themes, subthemes and nodes.

Three major themes were constructed from the data: i) negative impacts of falls alarms, ii) juggling the safety-risk conflict, and iii) negotiating falls alarm use. A summary of the themes, subthemes and nodes is presented in Fig 1.

### Theme 1: Negative impacts of falls alarms

Nurses’ negative experiences of falls alarms were related to noise and technological limitations. Nurses’ concerns and frustrations with the noise from falls alarms were multifaceted. The noise disturbed the patients in whom the falls alarm was in place, as well as other patients on the ward *“…the person has to hear the noise*, *and everyone else has to hear the noise (EN 11)”*. Nurses described sleep disturbance as a major issue and patients perceiving the noise as ‘high pitched’ and ‘horrible’. There was also additional frustration from being unable to moderate the noise without physically having to disarm the alarm at the bedside: “…*the alarm can’t be remotely monitored…” (Nurse 11—RN)* and “… *you can’t make it stop until someone goes there and stops it” (Nurse 12—RN)*.

The technological limitations of falls alarms described by were many nurses. False alarms were commonly triggered by slight patient movement such as *“…shifting in their seat…” (Nurse 1—EN)*, *‘…wriggling in bed…” (Nurse 2—ANUM) or “…simply breathing…” (Nurse 11 –EN)*. The sensitivity of the falls alarms, high number of false alarms, and noise resulted in both nurses and patients turning them off: “…*sometimes I know they [nurses]… turn it off for the meantime… you’re not supposed to” (Nurse 5—ANUM)* and “…*often the patients …stand up by themselves and turn them off*. *(Nurse 2—RN)*. Nurses also described problems with the falls alarm pagers. There were days where there were not *“…enough pagers to go around” (Nurse 9—EN)* so nurses left them at the nurses’ station, hoping someone would notice when one went off. There were mismatches where the falls alarm would activate but the pager did not, and known issues related to pagers not working when they were too far away from the alarm: “…*the pagers are only set up for about 50 metres” (Nurse 2—ANUM)*.

### Theme 2: Juggling the safety-risk conflict

Nurses described tension between using falls alarms to decrease the risk of a fall and falls alarms creating other risks. Falls alarms triggering agitation, particularly in patients with cognitive impairment who could not make sense of the noise, was a common experience. Nurses spoke about the noise from the falls alarm *“…panicking them [patients]…” (Nurse 6 –RN)*, *“…triggering behaviours…” (Nurse 6—RN)*, *and “…promoting aggravation*, *agitation…” (Nurse 10—EN)* thus the falls alarms were viewed as harmful: “…*making them [patients] worse … irritating them [patients] more…” (Nurse 8 –RN)*. The resultant patient distress and agitation was perceived to increase the risk of falling, rather than reducing falls risk as intended:

*…they’ve got delirium or dementia and are so anxious about that noise*. *That, to me is going to promote a fall rather than them just standing up and going for a walk (Nurse 12 –RN)*

*I think it [falls alarm] makes them more of a falls risk because they panic when they hear this beeping going off… they try and reach it to turn it off so it makes it a greater falls …*. *In those circumstances, I think they’re safer without it [falls alarm] (Nurse 9 –EN)*

The goal of care for patients on the study ward was rehabilitation and promoting independence in preparation for discharge. The use of falls alarms in these patients was counterintuitive and perceived as either harmful or ineffective by nurses. Nurses described that part of preparation for discharge was to allow necessary risk taking “…*we need to allow them to take a certain amount of risks … we really need to take the alarm away” (Nurse 9 –EN)* as patients would not have a falls alarm at home. Further, one of the unintended consequences of falls alarms was reduced mobility as patients were “…*not able to even move side to side or reposition without an alarm going off” (Nurse 10 –EN)* which was in direct opposition to the goals of reconditioning and increasing independence. Many nurses acknowledged that patients with falls alarms in place, still experienced falls “…*if they’re going to fall*, *the bed/chair alarm isn’t going to protect them … it’s not a protection*, *it’s just a warning (Nurse 11 –RN)*.

The need to respond to falls alarms frequently interrupted nursing care and nurses described the stress of balancing competing priorities and clinical risks. Several nurses described their experience of attending to intimate patient care for one patient and hearing a falls alarm activated for another patient. There was no one course of action that was not distressing for nurses. They could leave the patient they were attending to respond to the falls alarm, resulting in clinical risk and indignity for the patient:

*Mentally, that’s quite challenging… knowing that I’ve left my buddy standing there holding a patient that’s nearly naked with a towel over them while I’m trying to answer another bed chair alarm*. *(Nurse 12 –RN)*

Alternatively, nurses could stay with the patient for whom they are caring and wonder what was happening to the patient in whom the alarm was triggered:

*You might be in the bathroom attending to somebody*. *You can’t leave them to go and answer a bed/chair alarm that’s going off… you can’t leave that person… they could fall over. (Nurse 2 –ANUM)*

Several nurses perceived that falls alarms created clinical risk because whilst nurses were responding to falls alarms (many of which were false alarms), other patients missed nursing care. Nurses felt that “*… the time we spend running to the alarms*, *we can actually spend with the patients” (Nurse 6 –RN) and “…we have more people on alarms than we have nurses that can manage them” (Nurse 9—EN)*. As a result, patients with falls alarms received more attention at the detriment of other patients, or the time spent responding to falls alarms meant that missed care had to be handover over, both of which invoked feelings of guilt. The other source of clinical risk reported was interruptions to safety-critical tasks like medication management: “… *when you run to another patient …*. *you cannot remember which medications you have dispensed (Nurse 5 –ANUM)*.

### Theme 3: Negotiating falls alarm use

Falls alarm use was highly dependent on nurses’ decision making and many influences on nurses’ decisions regarding falls alarm use, such as years of experience, staffing levels, patient characteristics, family expectations and organisational policy, were associated with alarm overuse. There was a view that inexperienced nurses had a lower threshold for using a falls alarm “*… our new grads [graduate nurses] think all the confused patient has to be on a bed chair alarm” (Nurse 3—ANUM)* and that experienced nurses were more willing to “…*give them [patients] a chance” (Nurse 10—EN)*. There was also a sense that falls alarms were warranted overnight to compensate for reduced staffing: “…*[we need falls alarms] especially at night … there’s only three staff on” (Nurse 2—ANUM)*.

Patient characteristics were an important influence on nurses’ decisions to implement falls alarms. Nurses reported they were more likely to implement falls alarms if patients were “…*unsteady on their feet … not pressing the bell…” (Nurse 8 –RN)*, had cognitive impairment, or were unable to remember and follow instructions such as using the call bell or not getting up unattended: *“…it’s a combination of cognition*, *understanding instructions*, *and being able to do that safely*… *(Nurse 11—RN)*. A converse view was held by some nurses who considered it was better to manage falls risk in other ways, such as allowing safe wandering: “*… if they’re confused and want to wander in their room… you take the bed/chair alarms off …” (Nurse 2—ANUM)*. Family expectations often swayed nurses to use falls alarms and nurses felt that alarms were evidence to families that falls preventions strategies were in place:

*There’s an expectation … I tell the family we are trying our best to keep mum in a safe environment …if something happens to her despite of all the preventive equipment that we have in place*, *they are more understanding (Nurse 3—ANUM)*

Nurses talked about different levels of decision-making autonomy and the decisions to implement a falls alarm was seen as easier than the decision to remove the device:

*I can actually implement putting one on if there’s a need*. *I don’t feel as in control to actually say no, I don’t want one [a falls alarm] if someone is on one already … to remove it, I don’t think there’s as much autonomy (Nurse 8—RN)*

Similarly, nurses reported ‘inheriting’ alarm use in patients arriving from other areas: “…*from an acute hospital or sometimes from palliative care and they’ll be on a bed chair alarm” (Nurse 9—EN)*. Feeding into nurses’ perceived lack of decision making autonomy was the influence of organisational policy on their decisions to use or not use falls alarms. There were perceptions of reactive alarms use in response to a fall, particularly for falls with a clear explanation, and that falls alarms were a ‘box ticking’ exercise: “…*because she’d a fall*, *they decided that they’d tick the box” (Nurse 12—RN)* used for medico-legal reasons “…*probably a legal thing that nobody wants to be sued” (Nurse 9—EN)*.

## Discussion

The aim of this study was to explore nurses’ experiences of using bed and chair alarms. Three main themes were developed from the data: i) negative impacts of falls alarms, ii) juggling the safety-risk conflict, and iii) negotiating falls alarm use.

The negative impact of falls alarms in a subacute care setting was largely related to the disruptive nature of the noise associated with falls alarms and dissatisfaction with the technology. Nurses in our study reported that falls alarms were a significant source of noise on the ward, and the noise generated from falls alarms contributed to both sleep disturbance and increased agitation, particularly in patients with cognitive impairment. Hospitals are noisy environments and regularly exceed the World Health Organization’s recommended noise levels [19,20]. Noisy clinical environments are associated with sleep disturbance which increases risk of falls but also increases risk of delirium (which in turn increases falls risk) [20,21]. Further, falls with injury are less likely in quieter hospital environments [22]. The technological limitations of falls alarms are well documented, including the failure to discriminate between true and false positives [23]. A 2020 Australian study showed that false alarms (52%) were more common than true alarms (47%) and that in 43% of the alarms, staff were physically present with the patient [11].

Juggling the safety-risk conflict highlighted a complex interplay between the use of technology intended to enhance patient safety, and the unintended risks that technology created for patients in whom it was used, other patients, and nurses. Nurses in this study perceived that falls alarms exacerbated agitation, particularly in cognitively impaired patients which has been reported by several authors [24–26]. Nurses in our study also spoke about falls alarms limiting patient mobility, which created tension between falls prevention and promoting patient independence in preparation for discharge. The unintended consequences of reduced patient mobility with the implementation of falls prevention strategies is well-reported [5,27] and patients report that falls alarms confine them to bed [28]. Some authors contend that falls alarms are an unethical impairment of patient autonomy for free movement and encroach on patient dignity [24,25] and others have the position that falls alarms cause iatrogenic damage by preventing enhancement of mobility and core strength, thus increasing, rather than decreasing falls risk [29].

Interruptions to nursing care, and the associated risk of those interruptions was reported by several study participants. Nurses experience stress from leaving a patient requiring assistance with hygiene or toileting, interruptions to safety-critical tasks such as medication management, and from being unable to complete care such as dressings because of the time taken to respond to falls alarms (many of which were false alarms). The disruption of falls alarms to nursing care is documented in other studies [30–32] and nurses in other studies have also described the urgency to respond to falls alarms as source of increased stress and anxiety [30].

Finally, when considering falls alarm use, nurses described the many influences on their decision-making to use, or not use, falls alarms and a preference for more autonomy in implementation of falls alarms as part of the falls minimisation strategy to mitigate falls alarm overuse. Nurse characteristics associated with reducing falls include being confident in clinical decision making, having a formal (team leader or unit manager) or informal (ward expert) leadership role on the ward, and years of nursing experience [30]. Highly skilled nurses actively managed falls risk by correcting underlying causes such as hypovolaemia, reducing tethers, and actively managing medications [30].

There is evidence that clinical judgement is an appropriate way to inform falls prevention strategies. Despite widespread, and often mandated, use of falls risk prediction tools, their effectiveness in preventing falls in hospital patients is questionable [33–35]. It is also important to note that a patient’s falls risk is momentary and changeable thus requiring periodic reassessment [36]. In contrast, falls risk screening tools are static from one point in time and thus often cannot accurately predict falls risk [37].When compared to clinical judgement by a multidisciplinary geriatric team, the St. Thomas Risk Assessment Tool in Falling Elderly Inpatients (STRATIFY) did not exhibit superior performance in terms of classification accuracy (55% vs 48%) [38]. Further, clinical reasoning with a decision support list was not inferior to using a falls risk assessment tool to assign falls risk score and there was a trend towards reduced falls in the clinical judgement group [1].

The common default position of identifying patients at risk of falls, implementing falls alarms, and then responding to falls alarms has questionable effectiveness [30].The use of falls alarms by nurses as a dominant falls prevention strategy is predicated on the notion that rapid response to the audible alarm would prevent a fall [30]. However, several studies have shown that falls alarms are not effective in reducing falls or falls with injury [7,9]. A 2021 systematic review and meta-analysis of three randomised controlled trials involving 29,691 patients showed a 19% increase in falls among elderly patients with bed, bed-chair, or chair sensors in place (compared to no sensors) [23]. Overuse of falls alarms is therefore not without risk. There are many unintended consequences of falls alarms that may cause harm, which outweigh the likely negligible, theoretical benefits of using falls alarms.

There were limitations to this study that should be considered when interpreting the findings. Participants were a purposive sample of twelve nurses from one ward who agreed to participate, thus may have had a specific interest in falls prevention or use of falls alarms. Participants did vary in age, years of experience, level of appointment, and gender. The interviews were relatively short, likely the result of the semi-structured nature of the interview guide and very specific topic. That said, many nurses had similar experiences of using falls alarms, so the interview duration did not appear to impact on the study data. The findings from this study are not generalisable but provides insight into falls alarms use from a nursing perspective.

### Conclusion

Nurses’ experience of using falls alarms was predominantly negative and there was tension between falls alarms having limited impact on patient safety and risks associated with their use. Nurses described a need to support nurse decision making related to falls alarms use in practice and policy, and a desire to be empowered to manage falls risk in other ways.
